# COVID-19 Disparities in Nursing Homes

**DOI:** 10.3390/healthcare9040388

**Published:** 2021-04-01

**Authors:** Linda Loubert

**Affiliations:** Economics Department, Morgan State University, Baltimore, MD 21251, USA; linda.loubert@morgan.edu

**Keywords:** COVID-19 cases and deaths, nursing homes, disparities, the elderly

## Abstract

Cases of COVID-19, the coronavirus that has spread throughout the world, affect and kill the poor, racial minorities, and the elderly disproportionately. The toll to the elders of our society is extreme and does not seem to relent over time. This work examines the statistics of the cases and deaths at the early onset of the disease in nursing homes. It reveals the age disparities seen in the COVID-19 pandemic by using data from nursing homes across the states, and particularly in Maryland, as a source to illustrate the effect on the elderly living in group-like settings. It presents an overview of the disease in the early months as it ravaged across the states, indicating that the older generation was a lot more at risk than the general population. It is necessary to illustrate these disparities even as the numbers of infections and deaths are not static.

## 1. Introduction

Evidence on the SARS-CoV-2, the virus that causes COVID-19 disease, started on the west coast in the United States in late January and early February 2020. The Centers for Disease Control (CDC) reported that these early cases came from Wuhan, China, and Europe. By the end of February, the first death from COVID-19 had occurred in a nursing home in King County, Washington [1]. In early March 2020, the World Health Organization (WHO) advised that this highly contagious coronavirus disease, COVID-19, had reached a pandemic level. There were over 1000 cases and 38 deaths from COVID-19 in the United States at this time. It was not until mid-March that many states took drastic measures, such as “stay-at-home” orders, to curb the virus’s spread for the population.

Essential workers, however, such as those employed in nursing homes, continued to work and thus were more susceptible to the spread of the virus. Compared to the general population, minority workers in long-term care facilities represent a much higher percentage of these workers [2,3,4]. As COVID-19 began its surge throughout the United States, nursing home patients and workers felt its blow via cases of the disease and death from the disease. However, this work focuses primarily on the residents in the nursing homes.

To underscore the depth of the problem in nursing homes, the Washington Post [5] reported on 1 June 2020 that more than 25,000 nursing home residents died, and over 60,000 were infected from COVID-19. The virus has killed more than 400 staff during this time, and over 34,000 have been infected. Lawmakers took note of the high numbers of deaths occurring in nursing homes and sought remedies. A briefing from a Congressional subcommittee on COVID-19 reported over 40,000 Americans had died in nursing homes [6]. They noted that the nursing homes and other long-term facilities represented just 0.4% of the total population [6], yet, at the time of the briefing, this population accounted for approximately half of the deaths from COVID-19. This briefing also revealed no comprehensive plan for distributing personal protective equipment (PPE) for many of the workers, and many lacked the required and proper PPE [7].

Although the virus’s cases and deaths were starting to rise due to the spread of people’s infection acquired from their travels, an upward trend with nursing home patients being infected and dying due to this contagious disease had begun. It appeared that the virus was targeting the elderly and minority communities [8,9,10]. This phenomenon for the elderly population led to the question of whether the elderly or those who care for them should pay the price of death due to the COVID-19 transmission spread?

An article in the *New York Times* [11] on the racial divide of COVID-19 cases and deaths in nursing homes reported that the death toll reached approximately 20 percent of the nation’s death toll, with Blacks and Hispanics affected the most. Within the first few months of the pandemic, media outlets and influential medical journals [7,8,9,10,12] also reported how the novel coronavirus, COVID-19, affected people of color more than Whites. Chowkwanyun and Reed [13] warn that this disparity’s contextual aspect should be investigated immediately so that conscious and unconscious bias does not play a role in its reporting. For one, African Americans are disadvantaged by low socioeconomic status (SES) and chronic stress caused by racial discrimination, and a large proportion suffer from comorbidity diseases. They have a high rate of employment in low-paying jobs, such as those found in nursing homes. It is no surprise that nursing home populations are where the virus affects and kills a higher proportion of people. Another source of the disparity is that proper PPE was not available. Anecdotal evidence from the article reported that staffers in nursing homes were given raincoats to wear due to the shortage of PPE.

During the early months of the disease, the toll to our society’s elders appeared extreme and did not seem to relent. Using nursing home residents across the states and in Maryland helps to illustrate the effect on the elderly living in group-like settings. Data on cases and deaths are noted within the United States, but Maryland’s state websites on COVID-19 for the elderly in congregate living quarters present quickly downloadable data. Maryland’s data provide more details than many other states to understand the COVID-19′s spread.

Nursing homes were considered to have the “perfect storm” for transmitting this disease, ultimately resulting in death. Part of this problem is due to the residents’ close quarters and the comorbidities that older people can have. The Centers for Medicare and Medicaid Services (CMS) surveyed nursing homes on their safety protocol if and when COVID-19 infected the premises [14]. They also discovered that some nursing home staff lacked appropriate PPE and did not adhere to proper hand hygiene. The *Baltimore Sun* newspaper [15] reported that health regulators from the CMS fined some nursing homes in Maryland due to deficiencies that put staff and residents at risk during the COVID-19 pandemic. During the early days of the epidemic, proper PPE was in short supply across the nation as a whole, not just in nursing home facilities. As mixed messages about the longevity of the disease propagated throughout the country, a constant remained: the elderly, particularly those in nursing homes, continued to contract the virus and become ill as well as die from it.

## 2. Materials and Methods

It is the CMS government agency that provides oversight on nursing homes. Specifically, they offer the Nursing Home COVID-19 Public File, which includes data reported by all nursing homes to the Center for Disease Control’s (CDC) National Healthcare Safety Network (NHSN) tracking system. All nursing home data on COVID-19 goes within the Long-term Care Facility Module under NHSN. Some of the items in this module include resident impact and the staff and personnel at the facilities. The data reporting is required, but the data’s first input may not have been as accurate as needed while the facilities adjusted to the necessity and understanding of the reporting requirements. Nonetheless, this data was used in a geographical information system (GIS) to compare and analyze COVID-19′s effect on nursing home residents.

With the help of GIS, this work brings layers of information on nursing homes, the population in states, and COVID-19 cases and deaths to support the argument of disparities of the virus found in nursing homes [16]. GIS has a long history of providing insight into the “where” of many health situations [17], yet, few journals have literature on the extent of the virus’s impact through the lens of GIS. The literature continues to explain the impact of this pandemics’ phenomenon with its deadly and infectious effect on nursing home residents as time moves on.

**Figure 1:**
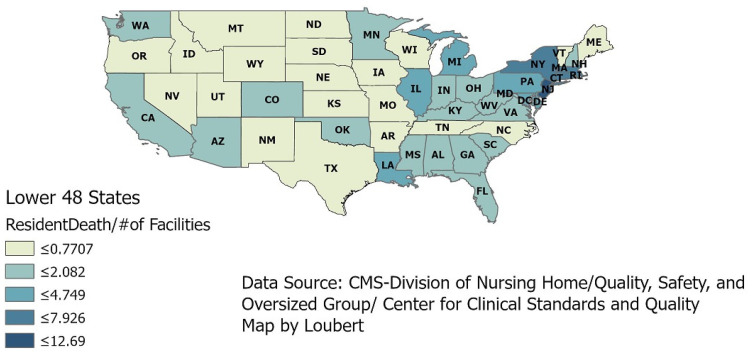
COVID-19 deaths of nursing home residents across the United States.

Using CMS’s data within GIS, a visual (Figure 1) of the continental U.S. presents the intensity of the deaths in nursing homes by showing deaths per the number of facilities within each state. The darkest areas represent the higher number of deaths per number of facilities, and it is in the eastern portion of the states during the early months of the pandemic where just this graphic shows more deaths.

Even though most information about this pandemic comes from paper and electronic news media, the data come from the Johns Hopkins Coronavirus Resource Center (CRC). This CRC continuously updated the sources of data on the virus. Although these data were produced within the first few months of the onset, this overall view helps us understand this pandemic’s severity. This slice of time gave a unique perspective of that period when the country tried to understand all the aspects of this raging pandemic. No actual policies were uniformly in place during the early months when these data were analyzed. When these data were obtained (31 May 2020), the highest number of deaths per facility was approximately 12 [14].

**Figure 2:**
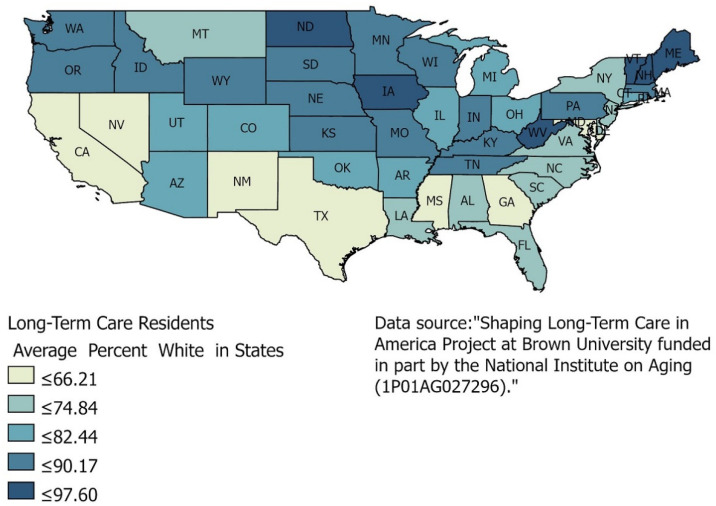
Percentage of White population in nursing homes.

Another source of data on nursing homes is the Brown University School of Public Health’s Long-Term Care: Facts on Care in the US. (LTCFocus.org) project. This project results from the Shaping Long-Term Care in America Project that Brown University Center for Gerontology and Healthcare Research produced. It is supported, in part, by the National Institute on Aging. LTCFocus.org hosts data regarding nursing home residents’ health and functional status, characteristics of care facilities, and other crucial long-term care issues. The data from LTCFocus.org provides an excellent source to understand many aspects of residents in nursing homes, particularly the residents’ racial diversity. The majority of the nursing homes are homogenous in their racial composition [8,12]. The map below (Figure 2) offers a glimpse of the racial diversity or the lack of diversity of nursing home residents. This map illustrates that the White population dominates the race of residents in nursing homes.

Racial data on the cases and deaths of COVID-19 by nursing homes was not available. Only aggregate reporting was done. That made it difficult to pinpoint where people of color outpaced White residents for COVID-19, even as the media reported that Blacks and poor people were being hit harder by the disease. Yet, over half of the states’ nursing homes are 89% or more White in population, and they are primarily the northern states where the higher percentage of the population is White. This GIS analysis helps understand who, to some extent, and where deaths occur due to the United States’ pandemic. South and Southwest states have a lower percentage of the White population, but they also have a higher Hispanic population rate, which the map does not show. Data found at the individual state level does not provide details on the race and gender of those who die from COVID-19. A better source is found at the county level in Maryland, even though CMS’s COVID-19 data can be aggregated at the county level. The State of Maryland provides an excellent source of online data that not all states offer, and therefore, its data is used in this work.

The Open Data website from Maryland [18] collects data by local health departments for Congregate Facilities, including nursing homes. The numbers of COVID-9-related cases and deaths in these datasets reflect totals ever reported in the health departments. As previously noted, the pandemic continues to spread and claim more lives. Therefore, this study only details the data up to the date when pulled from the source. This time does not mean a less accurate analysis is presented; it merely offers a snapshot of what was happening in nursing homes and how the elderly were disproportionately affected during the pandemic.

There are over 200 nursing homes in Maryland with a Black/White breakdown of 27 percent Black and 66 percent White. This sorting is similar to the states’ population racial breakdown of approximately 60 percent White, 30 percent Black, and 10 percent Hispanic. An exception that should be noted is two areas, Prince George’s County and Baltimore City, which have a majority Black population. The data collected from 15 April to 27 May on probable deaths from COVID-19 show that 51% are White and 32% are Black across the state and do not support any conclusion that more Blacks died in those two areas. However, the number of positive cases for the virus reveals that only 19% of Whites contacted the virus while 29% of Blacks and 25% of Hispanics contracted COVID-19.

**Figure 3:**
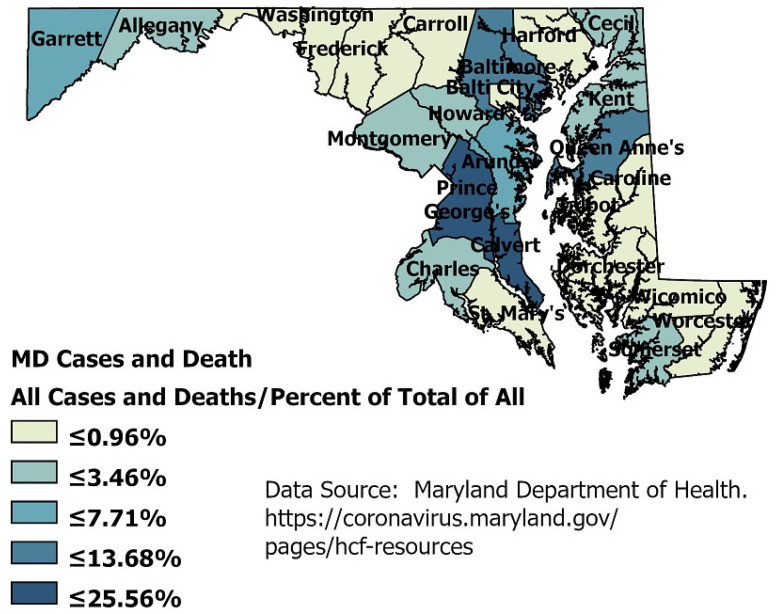
Counties in Maryland showing nursing home COVID-19 percentage of cases and deaths.

Racial data in nursing homes by county is not available, but the cases and deaths caused by COVID-19 can be seen across the state. Figure 3 shows all cases and deaths in nursing homes as a percentage of all cases and deaths for that county.

The county-level data in the map (Figure 3) does indicate that the elderly can bear the disease’s brunt with cases and deaths, even though the numbers of deaths range from 8 to 342 in certain areas. However, one county, Baltimore City, with the highest percent of Blacks, has a lower percentage of deaths in nursing homes than the county. Prince George’s county total deaths represent approximately one-third of all the state’s deaths, but those deaths are not occurring in the nursing homes. 

## 3. Results

**Figure 4:**
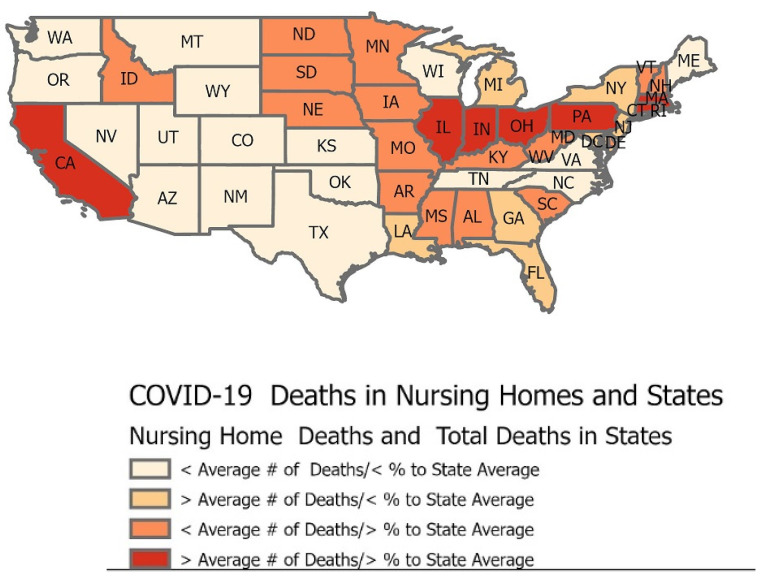
Map showing a comparison of the average number of COVID-19 deaths in nursing homes to the average number of COVID-19 deaths in each respective state.

A regression analysis of nursing home factors like race, gender, and age on deaths and cases would be a traditional method of analyzing the high numbers of COVID-19. This methodology could be used even as the numbers for these factors keep fluctuating daily. This GIS analysis performed by geocoding the data to the states provides insight outside of regression analyses as the numbers for the disease change. This data snapshot shown in the map below from 14 June 2020 is when the overall death numbers had surpassed 100,000. Figure 4 shows that only a few states had death rates from COVID-19 in nursing homes higher than the proportion of deaths from the state as a whole, but that in itself is significant, as nursing home residents represent such a small proportion of a state’s population.

A tabulation of the CMS data reveals that the average number of deaths among the states was 2252, while the average number of nursing home residents’ deaths was 604. The percent of a state’s nursing home resident deaths compared to the state’s total number of deaths ranged from a low of 10% to 57%, with an average of 32%. This statistic indicates that almost one-third of all of the virus’s deaths were occurring in nursing homes.

States clustering in the west portion of the states in the map in Figure 4 reveals that twenty of them (or 40%) have less than the average number of deaths of nursing home residents as a result of COVID-19, and these states have a smaller proportion of residents’ deaths compared to the overall number of deaths across the state. Seven states show deaths of nursing home residents from COVID-19 at higher numbers than the average, even as they had lower than average deaths across all state populations. These data show that the nursing homes in those states were hit harder for mortality. An indicator of disparity in deaths is those states (seventeen, or 17% of all states) whose nursing home deaths were smaller than the average of 604. Still, their proportion of fatalities in nursing homes was significantly higher than the average. Places like New Hampshire, Vermont, and Delaware had over 50% of their total deaths occurring in nursing homes. Even more alarming are those seven states whose numbers of deaths within the nursing homes were more than the average of 604 and had a larger than average share (>32%) of the deaths across the state population. Except for California, those states appear to cluster in the East.

The results of the states’ data trigger the notion that nursing homes can have high percentages of deaths compared to the general public, casting a harsh light on the fate of the elderly with this virus. Using data from the counties in the state of Maryland, one can see that deaths may even cluster within a state as perhaps the disease’s contagious nature fuels the death rate. Nearly one-third of the counties had 50% or more COVID-19 deaths in nursing homes, as shown in Table 1. The table below gives the number of deaths in nursing homes as well within the state by county.

**Figure 5:**
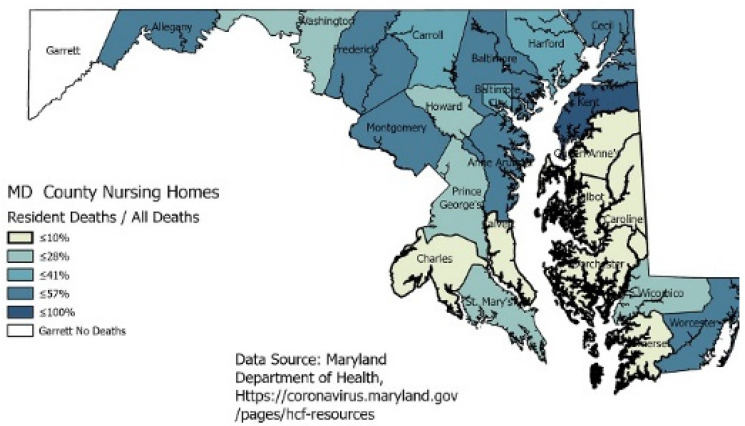
Map showing COVID-19 deaths in Maryland by county.

This table gives the number of deaths in nursing homes and in each county. By adding the percent of total deaths, it provides a perspective about those deaths. Adding the percent of residents who died from the virus compared to all of the county’s deaths shows how the disparities exist. It should be noted that nursing home residents represent less than 0.3% of Maryland’s population. This percentage may not be unlike most states. By mapping the data, the figure below (Figure 5) shows that the higher numbers of fatalities tend to be close in proximity. The highest number of nursing home deaths were also in the counties with the highest casualties due to COVID-19. These counties with the highest number of fatalities were Prince George’s, Montgomery, Baltimore City, and Baltimore County. Still, they did not necessarily have the highest percentages of deaths from nursing homes, as Table 1 presents. Nonetheless, approximately one-fourth of all the counties in Maryland had over 50% of deaths stemming from nursing homes.

## 4. Conclusions

As the data from this limited period indicates, the high percentage of nursing home deaths from COVID-19 is not just in counties with higher total deaths. A population representing a low proportion of the people within a state or county should not take on a more significant proportion of this disease’s deaths. This outcome is a travesty not only for the health care system but for the disparities it shows. It may take more research to fully understand why such disparities exist for the elderly in nursing homes. The data displayed in the maps point to a disparity for nursing home residents and not disparity according to race. As time passes and more researchers report their findings, aspects of the particular workforce in nursing homes may reveal how the virus can spread among the staff to the residents. Other studies may need to examine how the virus is fatal to the elderly with underlining conditions that accompany age. This study shows that the number of deaths due to COVID-19 affects nursing homes much more than the general population.

## Figures and Tables

**Table 1:** COVID-19 deaths in Maryland by county.

County	Resident Deaths	Deaths	Percent Nursing Home
Calvert	0	22	0.0
Caroline	0	2	0.0
Dorchester	0	4	0.0
Garrett	0	0	0.0
Queen Anne’s	0	14	0.0
Somerset	0	3	0.0
Talbot	0	4	0.0
Washington	6	24	25.0
Wicomico	8	37	21.6
Worcester	8	16	50.0
Allegany	9	17	52.9
Charles	9	84	10.7
St. Mary’s	9	44	20.5
Cecil	16	28	57.1
Howard	21	74	28.4
Harford	24	59	40.7
Kent	25	25	100
Carroll	36	106	34.0
Frederick	60	108	55.6
Anne Arundel	95	187	50.8
Baltimore City	97	301	32.2
Prince George’s	164	624	26.3
Baltimore County	202	415	48.7
Montgomery	342	667	51.3

## Data Availability

Data can be found at all the listed sites. The problem is that the data was pulled from websites and the data changed every day. The manuscript indicates when the data was pulled.
